# Community engagement and loneliness in older adults in China: mediation effects of social support in the wake of COVID-19

**DOI:** 10.3389/fpubh.2023.1199577

**Published:** 2023-12-01

**Authors:** Xiaoxia Xie, Chienchung Huang, Sophie Sitar, Xingyong Qiao

**Affiliations:** ^1^Research Institute of Social Development, Southwestern University of Finance and Economics, Chengdu, Sichuan, China; ^2^School of Social Work, Rutgers, The State University of New Jersey, New Brunswick, NJ, United States

**Keywords:** community engagement, loneliness, older adults, social support, COVID-19

## Abstract

**Introduction:**

Loneliness is a key indicator of well-being in older adults. Drawing from the ecological model of aging, the active aging perspective, and the convoy model of social relations, this study investigates the extent community engagement influences loneliness and whether the relationship is mediated by social support during the COVID-19 pandemic.

**Methods:**

Data was collected from 1,067 retired older adults in a cross-sectional design in Chengdu, China in 2022. Structural equation modeling was conducted to examine the direct and indirect effects of community engagement on loneliness through the hypothesized mediator of social support.

**Results:**

The results show community engagement was positively associated with social support (β = 0.26, *p* < 0.001) and social support was negatively related to loneliness (β = −0.41, *p* < 0.001). Social support fully mediated the relationship between community engagement and loneliness. Additionally, community engagement had an indirect effect on loneliness via social support (β = −0.11, *p* < 0.001).

**Discussion:**

The findings from the moderation analysis suggests community engagement and social support are likely to have large effects on loneliness for older adults over the age of 70 and who have low educational attainment. The findings suggest community engagement could be an important factor for improving social support and reducing loneliness amongst retired, older adults in China, especially in the wake of the COVID-19 pandemic where millions of individuals were isolated for extended periods of time.

## Introduction

Loneliness is a state of mind characterized by the perception of being alone and a discrepancy between desired and actual social relationships, regardless of the amount of social contact that an individual experiences (1). Loneliness has shown to be one of the key indicators of wellbeing in older adults. Empirical evidence has also shown that the experience of loneliness is related to poor health outcomes among older adults and can lead to conditions such as cognitive impairment, poor physical health, higher stress, suicidal ideation, and even depression (2–8). Moreover, approximately 16% to 30% of older adults report feeling lonely (9–12).

Loneliness has become a major concern in the medical field because of the detrimental outcomes it can have, and these concerns have only become exasperated in the wake of the COVID-19 pandemic (13). At the height of the COVID-19 pandemic, billions of individuals worldwide were forced to quarantine, stay-at-home, and isolate from their social networks and community to prevent the spread of the disease (7, 14). Given older adults tend to have more vulnerable health conditions, millions of older adults, particularly those who live in retirement homes or nursing homes, were forced into total separation from their families, communities, and even fellow residents (13, 15). This separation and isolation generated changes to loneliness for older adults regardless of gender and whether they live alone or with someone else (13). Because of its harmful impact on health and wellbeing, loneliness amongst older adults warrants rigorous examination to better understand how its effects could be mitigated during the pandemic. Such insights are crucial for improving our preparedness for future pandemics or catastrophic events.

Studies have also found the extent of loneliness in older adults varies by personal factors such as gender, stage of life, employment status, and health status, as well as environmental factors, such as number of people an individual lives with (3, 9, 10, 12, 16). For example, Srivastava et al. (10) found that retired individuals in India exhibited higher levels of loneliness (18.7%) than did working older adults (13.5%). Dong and Chen (16) found Chinese women had a higher rate of loneliness (28.3%) than older men (23.3%; *p* < 0.001). Additionally, Gierveld et al. (17) found age, marital status, and perceived lower health status (i.e., not as healthy as I could be) all are associated with higher levels of loneliness.

Regarding environmental influences, various factors such as family functioning, social networks, support systems, a sense of community, and geographic location have all been identified as protective factors against loneliness in older adults (10, 12, 18). The more social networks, emotional and physical support (10, 11, 18), familial interaction, and sense of coherence (6, 18), a person has, the lower the level of reported loneliness. Moreover, where a person chooses to live can also impact their perceived loneliness. For example, living in a rural area has a higher likelihood of loneliness than living in an urban area (10, 12). Additionally, the number of people within a household can also make a difference in loneliness (7). Earlier research shows people living alone reported higher levels of loneliness than do individuals living with at least one other person (13, 19).

Despite how significantly loneliness can impact health outcomes, most of the existing research on loneliness has been focused on the Western context, and less on community engagement and the mechanism between community engagement and loneliness (17, 19). The COVID-19 pandemic has also presented significant obstacles for older adults, including mental health difficulties and limitations in community involvement (7, 14). Given this, our study focuses specifically on the extent community engagement influences loneliness and whether the mechanism is mediated by social support amongst retired, older adults in Chengdu, China during the pandemic. Gaining insights into the interplay of these factors can assist policymakers, social workers, and administrators in senior care to improve the effectiveness of current active aging resources, support systems, and programs across China. The study's findings can also shed light on community engagement and loneliness amid the pandemic, thereby offering implications for addressing new surges of COVID-19 or potential future pandemics.

### Community engagement

Community engagement or participation refers the ways individuals are involved within their communities across various life domains, such as domestically, interpersonally, civically, and socially (20–22). By participating in community activities, individuals are provided with opportunities to establish connections and foster emotional support. Consequently, increased community engagement contributes to higher levels of connectedness and social support, which are associated with reduced feelings of loneliness and decreased psychological distress (23–28). That is, social support could serve as a mediator between community engagement and loneliness. For example, O'Mara-Eves et al. (20) conducted a meta-analysis of over 100 studies and found community engagement had a strong effect on social support (effect size=0.44) and self-efficacy (effect size=0.41). Additionally, Schwartz and Gronemann (26) found individuals with active participation in their communities had reduced levels loneliness (beta=-0.57).

Furthermore, in the context of older adults residing in China, the concept of community is frequently intertwined with familial support and connection. Research has demonstrated that these familial relationships play a crucial role in mitigating loneliness, particularly among older adults (15, 29). However, numerous facets of that cultural landscape have undergone transformations since the onset of the COVID-19 pandemic due to quarantine measures. These restrictions have impeded older adults from engaging with their communities and being in the company of their families and friends (14, 30). For example, during the height of the pandemic, the Chinese government implemented the zero-COVID strategy, which entailed imposing stringent lockdown measures. These measures effectively forbade residents from leaving their districts and imposed limitations on social and physical interactions. Individuals, including older, vulnerable adults, were forced to quarantine, stay-at-home, and isolate from their social networks and community to prevent the spread of the disease (14, 15, 30, 31). This separation and isolation generated changes to loneliness for older adults regardless of gender and whether they live alone or with someone else (7, 13).

As China attempted to gradually return to normal, it loosened the zero-COVID strategy by reducing the quarantine period and by allowing people to engage with normal business and community activities if zero COVID cases were reported in the district (32). For instance, in Chengdu, residents residing in low-risk areas were able to participate in regular activities, including community engagements like community reading and singing clubs. Notably, the city underwent a complete lockdown only once for a duration of 2 weeks in September of the year 2022 (33). Thus, it is important to examine the extent of community engagement on social support and loneliness during the pandemic given these changes.

### Social support

Social support is the perceived emotional and instrumental support individuals receive from others, including their family members, significant others, community members, and co-workers (24, 34, 35). Social support is associated with high levels of wellbeing and low levels of loneliness (36–39) because it enables positive self-esteem, companionship, and intimacy with others (40). Perceived social support can also impact an older adult's aging process and how he or she is able to transition to retirement or old age. For example, older adults who have poor or limited social supports tend to have negative attitudes toward aging which in turn can generate loneliness and depression (40). Kafetsios et al. (38) found perceived social support is negatively associated with loneliness (*r* = −0.53) for older adults in Greece. Kearns et al. (39) found the absence of emotional support (OR 1.68) and the lack of practical support (OR 1.54) were positively related to the extent of loneliness in the UK. Meanwhile, Chung and Kim (36) found social support was negatively related to loneliness (beta = −0.19) in Korea. Finally, Zhao and Wu conducted a study utilizing data from the China Health and Retirement Longitudinal Survey spanning the years 2013, 2015, and 2018. Their findings revealed that social support played a mediating role in the relationship between social participation and loneliness among older adults in China.

Retired, older Chinese adults have traditionally relied on their children or family members for social and community support (13, 15, 41). For many Chinese adults, it is of typical custom to live with their children through old age. However, due to China's one child policy, modernization of major cities, and changes in migration patterns for job opportunities, many retired, older adults are left living alone in rural areas without any familial social supports in place (41). Consequently approximately 30 million older adults live alone and this number is expected to near double by 2050 (42). Thus, retired, older adults can no longer only rely on their families for their social support and care (41). Instead, this support needs to come from the local community (37, 43).

In short, empirical data indicates that community engagement plays a crucial role in fostering social support and reducing levels of loneliness among older adults. It is worth noting that social support may act as a mediator between community engagement and loneliness. However, taking into account the influence of pandemic-related restrictions on community engagement (7, 13, 14), along with the significant and rapidly growing older-adult population in China (44), it is plausible that the changing ecological environment for older adults during the pandemic may exert dynamic effects on their levels of involvement in community engagement and social support. These changes, in turn, can affect their experiences of loneliness, aligning with the propositions of the ecological model of aging (45, 46). Thus, it is imperative to investigate the effects of community engagement on loneliness and the role played by social support in this relationship, particularly during the pandemic.

### Conceptual model and hypotheses

The ecological model of aging postulates the dynamic process of biological, behavioral, and environmental factors affects age progression (45–47). Moreover, this model indicates certain conditions and environmental factors can either promote or inhibit social connection and inclusiveness (48–50). Some of these conditions and factors include level of bodily functioning, confidence, self-esteem, family/friend relationships, proximity to social network, financial security, self-advocacy, access to community groups, and even access to politics (50). Of these factors, community engagement plays a significant role because it encourages individuals to actively participate socially, economically, culturally, and spiritually within their communities (27, 50). Ultimately, as the active aging perspective suggests, adequate community engagement can improve retired, older adults' quality of life (27), lower levels of loneliness, and even improved mental health and general wellbeing (23, 25, 27).

Additionally, according to the convoy model of social relations, relationships form an evolving social network that surrounds an individual and significantly influences their health and wellbeing throughout their lifespan (51, 52). This model emphasizes the reciprocal nature of support exchange between members of the social network, highlighting the importance of both giving and receiving support within the convoy. Moreover, the intrinsic significance of close social support relationships becomes particularly vital in later life due to the aging process, which diminishes the physical and mental capabilities of individuals to effectively cope with life's challenges in solitude (43, 52).

**Figure 1:**
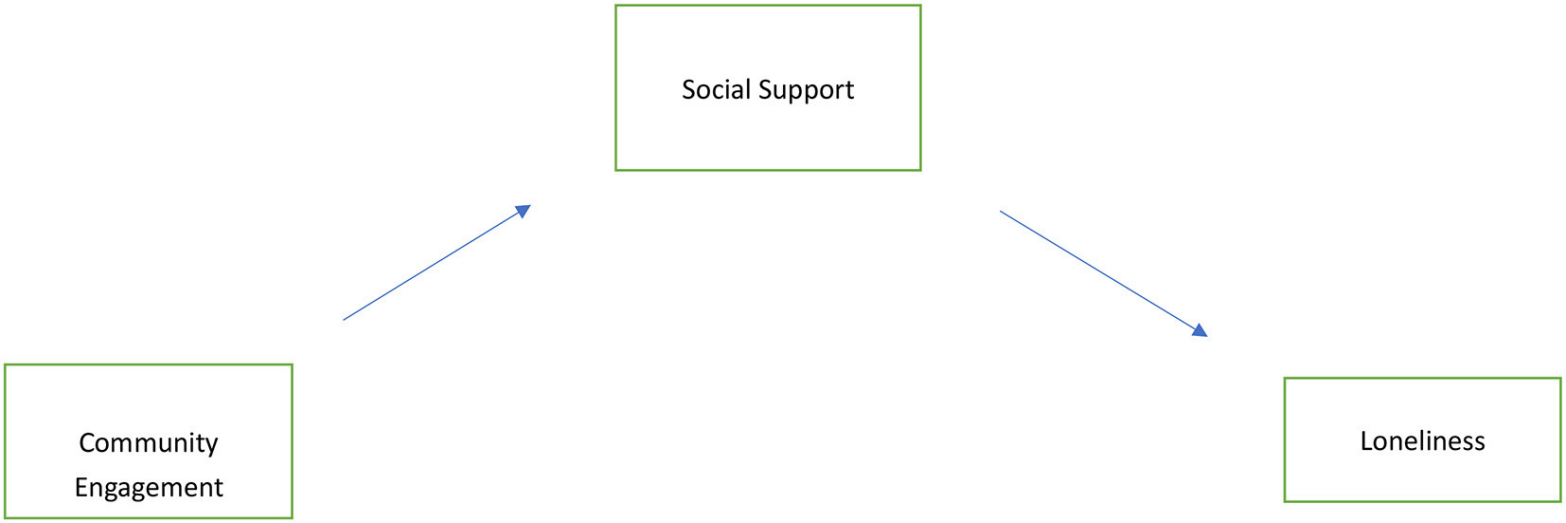
Conceptual model of community engagement, social support, and loneliness.

Based on the ecological model of aging, the active aging perspective, and the convoy model of social relations, this study aims to (1) examine the effects community engagement has on loneliness and to (2) investigate whether the effect is mediated by social support amongst retired, older adults in China. Additionally, we further examine whether these relationships are affected by personal factors such as gender, age, education, and living status. The conceptual model is illustrated in Figure 1. Specifically, we hypothesize:

(1) Community engagement is positively associated with social support.
(2) Social support is negatively associated with loneliness.
(3) Community engagement has a significant, indirect effect on loneliness via social support.
(4) The relationships amongst community engagement, social support, and loneliness were affected by personal factors such as gender, age, education, and living status.

Though current scholarship has shown community engagement has effects on loneliness via social support, there is little knowledge on social support's mediating effects between community engagement and loneliness, and how these two variables are influenced by personal factors amongst retired, older adults in China, especially in the wake of COVID-19. The findings of this study may contribute to the understanding of how community engagement affects loneliness through social support in a rapidly developing retired population and can shed light on potential policy and practices that may improve the outcomes of this population.

## Methods

### Data and sample

Our data collection was centralized to Chengdu, China retirement groups. We utilized a convenience sampling method to reach 1,167 seniors at different senior centers around the city from April 27 to June 27, 2022. Specifically, we chose senior centers based in five communities with high proportions of retired, older adults. These five communities were in low-risk COVID-19 zones, so residents were allowed to engage in normal business and community activities during the time of the survey. The inclusion criteria of the sampling process contained individuals who (1) resided within the five communities selected for this research, (2) were 50 years old or older, and (3) were retired at the time of the survey. We excluded older adults who never employed or were working at the time of the survey. Each community had around 250 retirees. Using the help of social workers and street-level social agencies, we distributed 1,167 questionnaires and received 1,085 completed back. However, 18 of the questionnaires were missing key variable data, so we removed them from the analysis. Thus, our total sample was 1,067 retired, older adults. Each adult was paid 3 RMB (0.5 USD) for their participation. Additionally, all participants were notified of their voluntary participation and given proper informed consent protocol prior to beginning the questionnaire. This research protocol was approved by the research review committee at the Research Institute of Social Development in the Southwestern University of Finance and Economics in China.

### Measures

#### Loneliness

We measured loneliness, the outcome variable, using the 8-item UCLA loneliness scale (“ULS-8”) (53). The ULS-8′s psychometric soundness, reliability, and validity have been previously verified in other studies performed worldwide (6, 31, 54, 55). Participants were asked to respond to different prompts such as “I lack companionship” and “There is no one I can turn to” and then rate how often they felt that way from 1 (“never”) to 4 (“always”). During our analysis, we reversed positive questions so higher scores indicated greater loneliness. Each participant was then given a total score ranging from 8 to 32. The Cronbach's alpha was 0.79.

#### Community engagement

Next, we measured community engagement using the 8-item community engagement scale, which has demonstrated acceptable reliability and validity within studies based on Chinese populations (56). Respondents reported the frequency they engaged in the following activities within their community: “meet and greet neighbors”, “ask neighbors for help”, “helping neighbors”; “discuss problems or issues in the neighborhood with neighbors”, “participate in community activities”, “report problems in the community to the management”, “participate in community meetings”, and “participate in community organizations”. The engagement formats contain both in-person and online approaches. Possible responses range from 1 (“never”) to 4 (“frequently”). Each participant's scores were then averaged. Possible scores ranged from 1 to 4. Here, the Cronbach's alpha value was 0.86.

#### Social support

Third, we measured social support using the 12-item perceived social support scale, (“PSSS”) (35) which has shown strong reliability and validity within studies based on Chinese populations (57–59). The PSSS assesses how participants perceive their social supports from family, friends, and others. For example, participants were asked to rate how strongly they agree from 1 (“very strongly disagree”) to 7 (“very strongly agree”) to statements like “I can count on my friends when things go wrong.” and “There is a special person in my life who cares about my feelings.” During our data analysis, we averaged the participants' answers and gave everyone a score ranging from 1 to 7. The PSSS had a Cronbach's alpha score of 0.95.

### Analytical approach

For all analyses we used version 16.0 of STATA statistical software. First, we performed a descriptive analysis of the key variables to understand the sample's characteristics. Second, we performed a Pearson's correlation analysis to investigate correlations amongst the variables. Third, we conducted a structural equation modeling (SEM) analysis to simultaneously examine the direct and indirect effects community engagement had on loneliness through the hypothesized mediator of social support. The SEM analysis was the preferred method because it allows for the simultaneous direct and indirect examinations of the mediating variable. The maximum likelihood (ML) estimation was used in SEM. We assessed the model-to-data fit using several fit indices, such as Chi-square statistics, Comparative Fit Index (CFI), Root Mean Square Error of Approximation (RMSEA), and Standardized Root Mean Square Residual (SRMR). Values of Chi-square statistics >0.0.05, CFI> 0.95, RMSEA values < 0.08, and SRMR < 0.08, all indicated good model-to-date fit. Finally, we further conducted the multi-group SEM analysis using personal factors, such as gender, age, education, and living status, to test the moderation effects (60). The multiple-group analyses provide the opportunity to investigate whether the estimated pathways within the model exhibit variations among different subgroups. These analyses involve the generation and comparison of subgroup estimates between an unconstrained model and a constrained model. In the unconstrained model, all estimated paths are allowed to vary across subgroups, while in the constrained model, all paths within the model are held equal across the subgroups. To assess the fit of these models to the data, we employ the likelihood ratio test. A significant test result indicates that the unconstrained model offers a superior fit to the data, suggesting that the pathways within the models differ across subgroups.

## Results

Table 1 lists the characteristics of the final sample. The average age of the sample was 65.9 years old (S.D. = 9.2), but a majority were between the ages of 60 and 69 (42.7%). Nearly one third of the sample was over 70 years old (32.1%) and one quarter were between ages 50 and 59 (25.2%). Additionally, 56% of the sample identified as female. About 44.4% of the sample reported having a high school education or higher, while 33.5% and 22.1% of them had only high school or below high school education, respectively. Finally, about 7% of the sample reported they lived alone, while 38.6% reported they lived with only one other person. However, most of the sample lived with more than 1 person (54.4%). The sample had an average community engagement score of 2.3. Perceived social support had a mean score of 4.9. Overall, the sample reported an average loneliness score of 15.8. Moreover, the descriptive statistics suggest on average the sample reported modest levels of community engagement, social support, and loneliness. The levels of community engagement, social support, and loneliness were varied by demographics as shown on Table 2.

**Table 1:** Descriptive statistics of key variables.

	**Mean (S.D.)**
Age	65.9 (9.2)
50–59 [%]	25.2
60–69 [%]	42.7
70 and above	32.1
Gender (Female) [%]	56.0
Education	
Below high school [%]	22.1
High school [%]	33.5
Above high school [%]	44.4
Living Status	
Alone [%]	7.0
With another person [%]	38.6
With more than one person [%]	54.4
Community Engagement [1–4]	2.3 (0.6)
Social Support [1–7]	4.9 (1.1)
Loneliness [8–32]	15.8 (4.2)

N = 1,067.

**Table 2:** Community engagement, social support, and loneliness by demographics.

	**Community engagement**	**Social support**	**Loneliness**
All	2.29 (0.59)	4.91 (1.05)	15.78 (4.16)
**Age**			
50–59	2.22 (0.60)	4.85 (1.12)	16.27 (4.23)
60–69	2.29 (0.54)	4.95 (0.97)	15.38 (4.09)
70 and above	2.35 (0.64)	4.89 (1.12)	15.93 (4.15)
F-test	10.6 ^**^	0.9	4.2 ^*^
**Gender**			
Male	2.33 (0.59)	4.91 (1.00)	15.75 (4.02)
Female	2.26 (0.58)	4.90 (1.11)	15.82 (4.27)
F-test	3.3	0.1	0.1
**Education**			
Below high school	2.28 (0.53)	4.66 (1.13)	16.71 (3.82)
High school	2.26 (0.61)	4.93 (1.11)	15.99 (4.14)
Above high school	2.32 (0.60)	5.01 (0.96)	15.16 (4.23)
F-test	1.2	9.1 ^***^	11.8 ^***^
**Living Status**			
Alone	2.29 (0.56)	4.88 (1.09)	16.83 (3.89)
With another person	2.30 (0.58)	4.89 (1.08)	15.63 (4.22)
With more than one person	2.29 (0.60)	4.91 (1.03)	15.76 (4.13)
F-test	0.1	0.1	2.7

N = 1,067. Numbers in parentheses show standard errors. ^**^p < 0.01, ^***^p < 0.001.

Table 3 presents the findings from the correlation analysis on the key variables. The findings were consistent with our hypotheses. First, community engagement had a positive correlation with perceived social support (r=0.26, *p* < 0.001) and a negative correlation with loneliness (*r* = −0.13, *p* < 0.001). Moreover, social support had a negative correlation with loneliness (*r* = −0.31, *p* < 0.01).

**Table 3:** Correlation analysis of key variables.

	**1**	**2**	**3**
1. Community engagement	—		
2. Social support	0.26^***^	—	
3. Loneliness	−0.13^***^	−0.41^**^	—

N = 1,067. ^**^p < 0.01, ^***^p < 0.001.

**Figure 2:**
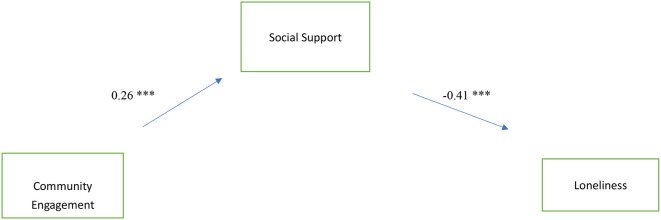
Standardized estimates of community engagement, social support, and loneliness. ^***^p < 0.001.

The standardized estimates of the SEM model are listed in Figure 2. The model fit statistics showed the proposed model fit adequately into the data: χ2 (1) = 0.67, *p* > 0.05, CFI = 1.00, RMSEA = 0.00, SRMR = 0.01. Additionally, community engagement had a positive effect on social support (β = 0.26, *p* < 0.001). Together, these results confirm Hypotheses 1. Consistent with Hypothesis 2, social support had a direct and negative effect on loneliness (β =−0.41, *p* < 0.001). The SEM analysis yielded results indicating that social support served as a complete mediator between community engagement and loneliness. This was evidenced by the satisfactory fit of the conceptual model, and it was found that community engagement exerted a significant indirect influence on loneliness through its effect on social support (β =−0.11, *p* < 0.001). These findings are consistent with Hypotheses 3.

The moderation analysis results are listed in Table 4. The results of the likelihood-ratio tests showed the estimates of community engagement on social support and loneliness were significantly moderated by age, education, and living status. The effects of community engagement on social support tend to be larger for adults who were over the age of 70, and between the ages of 50 and 59, with below high school level education, and who lived with more than 1 person. In contrast, the effects of community engagement were small for older adults aged between 60 and 69, with a high school education, and who lived alone. Social support had a larger effect on loneliness for older adults who were 60 years old and older, with an above high school level education, and who lived with another person. Overall, the indirect effects of community engagement on loneliness via social support tends to be large for adults over the age of 70, with a below high school level education, and who lived with 1 or more person.

**Table 4:** Direct and indirect effects of community engagement and social support on loneliness.

**Independent variable**	**Dependent variable**	**Direct effect**	**Indirect effect**
**All sample**			
Community engagement	Social support	0.26^***^	—
Community engagement	Loneliness	—	−0.11^***^
Social support	Loneliness	−0.41^***^	
**Gender**			
**Male**			
Community engagement	Social support	0.27^***^	—
Community engagement	Loneliness	—	−0.11^***^
Social support	Loneliness	−0.42^***^	
**Female**			
Community engagement	Social support	0.26^***^	—
Community engagement	Loneliness	—	−0.10^***^
Social support	Loneliness	−0.40^***^	
Likelihood-ratio test		8.45	
**Age**			
**50–59**			
Community engagement	Social support	0.30^***^	—
Community engagement	Loneliness	—	−0.10^***^
Social support	Loneliness	−0.33^***^	
**60–69**			
Community engagement	Social support	0.17^***^	—
Community engagement	Loneliness	—	−0.08^***^
Social support	Loneliness	−0.47^***^	
**≥70**			
Community engagement	Social support	0.33^***^	—
Community engagement	Loneliness	—	−0.14^***^
Social support	Loneliness	−0.41^***^	
Likelihood-ratio test		29.5^**^	
**Education**			
<**High school**			
Community engagement	Social support	0.41^***^	—
Community engagement	Loneliness	—	−0.15^***^
Social support	Loneliness	−0.36^***^	
**High school**			
Community engagement	Social Support	0.17^***^	—
Community engagement	Loneliness	—	−0.07^**^
Social support	Loneliness	−0.38^***^	
> **High school**			
Community engagement	Social support	0.27^***^	—
Community engagement	Loneliness	—	−0.12^***^
Social support	Loneliness	−0.44^***^	
Likelihood-ratio test		66.2^***^	
**Living status**			
**Alone**			
Community engagement	Social support	0.17	—
Community engagement	Loneliness	—	−0.04
Social support	Loneliness	−0.24^*^	
**With another person**			
Community engagement	Social support	0.22^***^	—
Community engagement	Loneliness	—	−0.11^***^
Social support	Loneliness	−0.51^***^	
**More than 1 person**			
Community engagement	Social support	0.31^***^	—
Community engagement	Loneliness	—	−0.11^***^
Social support	Loneliness	−0.36^***^	
Likelihood-ratio test		22.4^*^	

N = 1,067. ^*^p < 0.05, ^**^p < 0.01, ^***^p < 0.001.

## Discussion

The retired, older adults in this study, on average, reported moderate levels of loneliness. The average score of loneliness here was 15.9, which is lower than retirees report in Nigeria [20.3; (61)], but higher than what senior citizens report in the Philippines [7.2; (62)], higher than senior citizens in rural parts of China [11.1; (63)], higher than migrant older adults in China, [12.8; (31)], and higher than community older adults in Singapore [14.4; (64)]. These comparisons suggest that the degree of loneliness experienced by retired, older adults in Chengdu, China falls within the spectrum of loneliness observed in different studies focusing on older adult populations, both internationally and domestically.

The sample also reported modest levels of community engagement and social support. Although there is a positive inclination toward observing older adults engaging in community activities and receiving social support during the pandemic, evaluating the actual effects of the pandemic on the extent of community engagement and social support presents difficulties. This is primarily because the available data is derived from a one-time cross-sectional survey, which limits the ability to track longitudinal changes and draw definitive conclusions about the impact of the pandemic on these factors. Additionally, these descriptive findings are also limited to the experience of older, retired adults in Chendgu, China and may not be generalizable to individuals in other regions of China. Future research should expand upon our findings to investigate the extents of community engagement, social support, and loneliness among retired, older adults in either other Chinese cities or on a different time point to assess the effects of region and time on the statistics.

Next, the SEM analysis results showed community engagement had a modest effect on increasing social support and that social support had a strong effect on reducing loneliness during the pandemic. The findings indicate that social support plays a crucial mediating role between community engagement and loneliness among retired older adults in China during the pandemic. Moreover, these findings are consistent with the ecological model of aging, the active aging perspective, and the convoy model of social relations, and suggest that community engagement has the potential to improve quality of life by increasing social support and reducing loneliness (15, 23, 25, 43), and that social support, as a mediator, effectively reduces the extent of loneliness even during the pandemic (37, 43).

The findings of this study have several practical implications. First, given community engagement is significantly associated with social support and loneliness of retired, older Chinese adults, it is imperative for retiree programs to implement interventions and services that promote community engagement. These efforts will aid retired older adults in enhancing their social support networks and subsequently mitigating feelings of loneliness, especially in the face of new waves of COVID-19 or potential future pandemics. Although the Chinese government has made efforts to promote community engagement amongst older adults in recent years (29, 65) more efforts need to be taken to continue to improve community connections and residents' wellbeing given the findings of this study over the pandemic period. Doing so can increase health incomes for older adults in a major way when they face life challenges such as the global pandemic or other crises.

Second, given social support strongly effects loneliness, other interventions and services that are successful at improving social support should be implemented. For example, studies have shown social connection and network interventions can improve social support and reduce loneliness in older adults (66, 67). In addition, there is evidence that information and communication technology can improve life and social support for older adults (31, 68, 69). Consequently, social connection and advancing technology hold promises in improving social support amongst retired, older adults.

Third, the moderation analysis findings suggest community engagement and social support programs may work effectively for some groups, while have limited effects on others. Specifically, community engagement is likely to have large effects on social support for vulnerable groups such as older adults aged 70 and above (beta = 0.33) and with a below high school level education (beta=0.41). Thus, government agencies should prioritize targeting these groups for interventions and services to improve their perceived loneliness during the pandemic or other crises.

However, for older adults who live alone, community engagement seems have a small effect on social support and that social support also has limited effects on loneliness. These findings suggest community engagement might not strongly related to social support and loneliness of older adults living alone. The findings are in line with previous research on community engagement, social support, and loneliness for older adults living alone (19, 70). For example, Schafer et al. (19) found for Americans and Europeans, having larger, diverse community networks outside the home reduces the loneliness of living alone, but even still with extensive community connections, individuals who live alone report higher levels of loneliness than those who live with someone else. Thus, the Chinese government and various social work agencies may want to seek other approaches such as technology or peer-based interventions to increase social support and reduce the extent of loneliness for older adults who live alone (68, 71). For example, Czaja et al. utilized a randomized field trial and found that the communication technology intervention was significantly increased social support and reduced loneliness for older adults living alone.

This study also has several limitations. First, the study used a cross-sectional design, which prevented us from inferring any causal relations among community engagement, social support, and loneliness. Future studies should consider implementing a longitudinal design to account for temporal sequencing and to better understand the causal relations among these variables. In particular, the design should include both pandemic and post-pandemic period to comprehend the effects of the pandemic on these variables. Second, the data relied on self-reports of retired, older adults in Chengdu, China. Though self-reporting is a common method for data collection, it might be associated with self-reporting biases that can affect the estimates of the results. Third, the data collection occurred in one city, Chengdu, so our findings may not be generalizable to the larger population of retired, older adults in China. A future study could expand upon our findings by examining how geographic differences in China (e.g., rural vs. urban) might affect the mediational pathway between community engagement and loneliness through social support. Also, the sample is limited to retired, older adults, so the findings may not be generalizable to older adults who never had a job or who were working part time after retirement.

Fourth, the results demonstrate the effects of community engagement on social support tend to be greater for older adults over the age of 70 that live with at least one other person. However, our study primarily consisted of individuals under the age of 70 (the average age of the group was 65.9 with a standard deviation of 9.2). Thus, future research may want to focus on the experience of individuals who are living with at least one other person and are over the age of 70 to understand specifically what aspects of community engagement and social support associate with their loneliness most. Moreover, individuals who retire are at higher risk for developing depression and other mental disorders because of the major social changes and financial challenges retirement can bring (72). Thus, newly retired individuals who are still going through this major life transition, may have skewed perceptions of their loneliness and social connection with others. Given our sample was particularly young and just about at the age for retirement in China, participants may have skewed answers about their perceived loneliness because they are still adjusting to newfound life and social dynamics. Future research may consider assessing the perceptions of retirees who have had many years to adjust to their realities and support networks.

Fifth, we found community engagement has limited effect on social support (β = 0.17) and social support has no significant effect on loneliness for old adults living alone. This could mean that community engagement during the pandemic appears to have small effect on social support and loneliness among older adults living alone. However, the sample size for this group was relatively modest, comprising only 7% of the sample (*n* = 75). Consequently, additional research is required to gain a comprehensive understanding of how community engagement affects social support and loneliness among older adults living alone, using larger and representative samples.

Finally, the COVID-19 pandemic changed the way people are able to interact with their community and social support systems. For example, research shows more older adults in China are acquiring access to technology, such as the internet and smartphones, to facilitate communication and connection (69, 73, 74). This study does not incorporate how technology influences loneliness, community engagement, and social support. Future research should explore how these tools can help older adults reduce loneliness and build connection. Moreover, while this study recognizes the impact COVID-19 has had on loneliness amongst older adults in China, the study does not seek to understand how the pandemic changed loneliness. Instead, it merely examines how loneliness is now for older adults in Chengdu, China without comparing it to what loneliness levels may have once been. While there have been some studies on the impact COVID-19 has had on loneliness (13), future studies should continue to explore the long-term effects COVID-19 has had on older adults in Chengdu, China and understand how different interventions may work to improve loneliness, social support, or community.

## Conclusion

In a sample of 1,067 retired, older Chengdu, China based adults, there was evidence community engagement influences loneliness, and this relationship is mediated by social support during the COVID-19 pandemic. Moreover, these findings support existing cross-cultural research on community engagement, social support, and loneliness. Community engagement should continue to be used as a mechanism to improve social support and to ultimately reduce loneliness. Loneliness has a powerful effect on individuals and can negatively affect general health and wellbeing of individuals, particularly for retired, older adults, during the pandemic and other life crises. This study calls for interventions and services that promote community engagement and social support for retired, older adults in China. This is incredibly imperative, given the COVID-19 pandemic has isolated many older adults from necessary community and social supports, and continues to have a lasting impact today.

## Data availability statement

The raw data supporting the conclusions of this article will be made available by the authors, without undue reservation.

## Ethics statement

The studies involving humans were approved by the Research Review Committee, Research Institute of Social Development, Southwestern University of Finance and Economics. The studies were conducted in accordance with the local legislation and institutional requirements. The participants provided their written informed consent to participate in this study.

## Author contributions

XX and CH: conceptualization and resources. XX, CH, SS, and XQ: methodology and software, validation, formal analysis, and writing—original draft preparation. XX, CH, and XQ: investigation and data curation. All authors contributed to the article and approved the submitted version.
